# Mice with cancer-induced bone pain show a marked decline in day/night activity

**DOI:** 10.1097/PR9.0000000000000614

**Published:** 2017-08-11

**Authors:** Lisa A. Majuta, Jean-Marc G. Guedon, Stefanie A.T. Mitchell, Michael A. Kuskowski, Patrick W. Mantyh

**Affiliations:** a Department of Pharmacology, University of Arizona, Tucson, AZ, USA; b Department of Psychiatry, University of Minnesota, Minneapolis, MN, USA; c Cancer Center, University of Arizona, Tucson, AZ, USA

**Keywords:** Bone, Pain, CIBP, Functional status, Activity, Day/night activity

## Abstract

**Introduction:**

Cancer-induced bone pain (CIBP) is the most common type of pain with cancer. In humans, this pain can be difficult to control and highly disabling. A major problem with CIBP in humans is that it increases on weight-bearing and/or movement of a tumor-bearing bone limiting the activity and functional status of the patient. Currently, there is less data concerning whether similar negative changes in activity occur in rodent models of CIBP.

**Objectives:**

To determine whether there are marked changes in activity in a rodent model of CIBP and compare this to changes in skin hypersensitivity.

**Methods:**

Osteosarcoma cells were injected and confined to 1 femur of the adult male mouse. Every 7 days, spontaneous horizontal and vertical activities were assessed over a 20-hour day and night period using automated activity boxes. Mechanical hypersensitivity of the hind paw skin was assessed using von Frey testing.

**Results:**

As the tumor cells grew within the femur, there was a significant decline in horizontal and vertical activity during the times of the day/night when the mice are normally most active. Mice also developed significant hypersensitivity in the skin of the hind paw in the tumor-bearing limb.

**Conclusion:**

Even when the tumor is confined to a single load-bearing bone, CIBP drives a significant loss of activity, which increases with disease progression. Understanding the mechanisms that drive this reduction in activity may allow the development of therapies that allow CIBP patients to better maintain their activity and functional status.

## Abstract 2

Supplemental Digital Content is Available in the Text.

Mice with bone cancer have reduced peak day/night activity. Therapeutically blocking this decline may improve the activity level of patients with painful bone metastases.

## 1. Introduction

Cancer-induced bone pain (CIBP) is a unique pain state with overlapping but distinct features including mechanical weakening of the tumor-bearing bone as well as generation of both an inflammatory and neuropathic pain.^18,19,33,42,44,45^ Early in the disease, the most notable change is usually an alteration in the balance of bone destruction and bone formation.^13,23,39^ Importantly, even in tumors such as prostate where both bone destruction and bone formation occur, the newly formed “woven bone” is mechanically weak, fractures much more easily than healthy bone, and excessive bone destroying osteoclast activity is prevalent.^30,35,69,70^ Thus, with both osteolytic (breast, lung, renal, sarcoma, and myeloma) and osteoblastic (prostate) tumors, there is generally a marked weakening of the tumor-bearing bone such that normally innocuous loading or palpation of the bone will activate the mechanosensitive nociceptors that innervate the bone.^32,43,61^

A second process that drives CIBP is increased sensitization and excitation of bone nociceptors that are driven by both cancer cells and their associated stromal cells. As tumor cells invade bone, both size and numbers of osteoclasts can increase dramatically, which not only destroys bone but also induces a highly acidic environment.^12,24,32,64^ This acidic environment, as well as the release of inflammatory mediators by cancer and associated stromal cells,^29,53,58,63^ both sensitizes and activates nociceptors resulting in both an ongoing and movement evoked pain.^32,61^

The third component of CIBP is neuropathic. As the tumor colonizes and remodels the bone, the distal ends of the nerve fibers that normally innervate the bone marrow, mineralized bone and the periosteum are destroyed.^43,52^ Following this initial destruction, factors released by cancer cells and their associated stromal cells can also induce an exuberant sprouting of the remaining, damaged nerve fibers leading to a hyper-innervation of sensitized nociceptors in the marrow, mineralized bone, and periosteum.^4,38,47^ This ectopic nerve sprouting results in an increase in the density of highly sensitized mechanosensitive nerve fibers, even in areas of cortical bone that are normally poorly innervated.^46^ Now, any mechanical strain and/or distortion of this weakened bone will presumably be detected by this increased number of sensitized mechanoreceptive sensory nerve fibers, resulting in normally innocuous movement and loading of the bone being perceived as a highly noxious event.^32,61^

Two decades ago, the average life expectancy of a patient with bone cancer could usually be measured in months and the major emphasis was simply to relieve pain.^3,67^ Now the same patient may live years and even decades.^3,9,37,62,67^ Given this change, maintenance of the activity and functional status of the patient has become a high priority.^3,39^ Here, this issue is explored in a rodent model of CIBP combined with continuous day/night monitoring using automated activity boxes.

## 2. Methods

### 2.1. Experimental animals

Experiments were performed on 50 adult, male C3H/HeJ mice (Jackson Laboratories, Bar Harbor, ME), approximately 8 to 9 weeks old at the beginning of the experiment, weighing 22 to 30 g at the time of surgery. Ten mice were used to establish the effect of the sarcoma surgery itself (no cells injected) on activity and rearing. Forty mice were used to establish the timeline and effect of sarcoma on activity, rearing and tactile sensitivity (25 sarcoma and 15 naive). Mice were randomly placed in groups and ear-tagged prior to any testing or surgical procedures. Animals were individually housed (AAALAC approved SPF facility, Lab Products IVC 750 cages, 6.75″ × 12.25″ × 5″, with ¼″ corn cob bedding and nestlet) at least 1 week before baseline recordings and continued throughout the duration of the experiment. Mice were housed in accordance with National Institutes of Health Guidelines and kept in a vivarium maintained at 22°C with a 12-hour alternating light–dark cycle and provided food and water ad libitum. All procedures adhered to the guidelines of the Committee for Research and Ethical Issues of the International Association for the Study of Pain and were approved by the Institutional Animal Care and Use Committee at the University of Arizona (Tucson, AZ, USA).

### 2.2. Cancer cells

Osteolytic sarcoma cells, NCTC 2472, stably expressing GFP were cultured in NCTC135 media (Sigma, St. Louis, MO, cat# N3262) containing 10% horse serum (HyClone; Sigma), 50 mg Geneticin (Gibco; ThermoFisher, Pittsburgh, PA, cat# 10131-035), and 2.4 g sodium bicarbonate (Sigma, cat# S5761). For implantation into animals, cells were trypsinized (0.025% trypsin) off of flasks, centrifuged at 1500 RPM for 5.5 minutes, and resuspended in 1× HBSS (Gibco) at a concentration of 10 × 10^6^ cells/mL.

### 2.3. Surgery and the injection and confinement of cancer cells to the femur

As described previously,^48,57^ an arthrotomy was performed after induction of general anesthesia with ketamine/xylazine (100 mg/kg ketamine and 10 mg/kg xylazine; s.c.), a 1-cm incision was made in the skin overlying the knee on the lateral aspect, parallel to the femur. The skin over the knee was reflected, and the joint was exposed by transposing the patella medially after blunt dissection through the lateral parapatella tissues with the knee in flexion. A 0.5-mm diameter hole was drilled in the center of the trochlear groove of the femur using a pneumatic dental high-speed hand piece, avoiding the cruciate ligaments. A pin was inserted into the intramedullary canal to core the marrow space. Then 5 μL HBSS containing 5 × 10^4^ osteolytic murine sarcoma cells were injected into the intramedullary space. For the 10 mice used to test the effect of the surgery itself, no cells were injected into the femur. The drill site was sealed with a dental amalgam plug (Dentsply, Milford, DE). The knee was extended and the patella returned to its normal position in the trochlear groove. To minimize medial patella luxation, the fascia of the vastus muscles and the parapatella tissues near the knee were secured back in position using a horizontal mattress suture before the closure of the skin. Wound closure was achieved with two 7-mm auto wound clips (Becton Dickinson, Sparks, MD). Animals recovered from anesthesia on heating pads and received injections of antibiotic (Baytril, 85 mg/kg, s.c.) and sterile saline (1 mL, s.c.). After recovering from surgery, animals remained individually housed to avoid animals fighting, which increases the likelihood of displacement of the patella. The naive group of mice remained naive throughout the experiment.

### 2.4. Exclusion criteria

Animals could be excluded from the experiment under 4 conditions; surgical complications, a loss of more than 20% of their pre-surgery weight, if patella displacement had occurred or if no cell growth was evident; the latter 2 as identified through radiography. Two mice were removed from the sarcoma group due to patellar displacement (final n for sarcoma group = 23). One mouse was removed from the surgery test group due to patellar displacement (final n = 9).

### 2.5. The organization of the behavioral testing

Spontaneous locomotion and rearing were recorded over the 20 hours just prior to the animals being placed in the tactile sensitivity testing apparatus. All naive and cancer mice were acclimatized to the tactile sensitivity testing apparatus for 30 minutes on 4 consecutive days 1 week prior to naive baseline behavioral testing. All tactile behavioral testing was performed in the morning and completed before noon. Testing was performed at baseline (pre-sarcoma inoculation) and 7, 14, 21, 28, and 35 days postsarcoma inoculation. Spontaneous locomotion and rearing testing for the surgery test group were performed at baseline (pre-surgery) and 1, 3, 7, and 12 days postsurgery. Each individual behavioral measure was performed by the same experimenter for the duration of the experiment.

### 2.6. Assessment of horizontal and vertical activity

Mice were assessed for horizontal activity (distance traveled, cm) and the number of rearing episodes (vertical activity), which requires hindlimb loading.^40^ Animals were placed individually in plexiglass boxes (16 × 16 × 11.75 inches) containing a thin layer of bedding and a 1-inch square of Napa Nectar (Systems Engineering, Napa, CA). Horizontal and vertical activities were assessed via open field monitoring by arrays of photobeam sensors that use beam breaks to determine the location of each animal at all times (Omnitech Electronics, Columbus, OH) (Supplemental Figure 1, available online at http://links.lww.com/PR9/A7). Horizontal locomotor activity and the number of rearing episodes were continuously monitored for 20 hours beginning at 1200 hours (noon) in a light- and temperature-controlled testing room that remained closed to any other activity. Fusion software (Omnitech Electronics) was used to analyze and store the above parameters.

### 2.7. Cutaneous stimulus–evoked pain (von Frey test)

Skin pain (tactile sensitivity) was assessed using calibrated von Frey filaments (Stoelting, Wood Dale, IL) and using the up-down method (Chaplan, Bach et al. 1994). Von Frey filaments, beginning at 0.4 g, were applied in the ascending order (log scale) to the mid-plantar surface of the hind paw. The time between filament applications was at least 5 seconds. Fifty percent withdrawal thresholds were calculated by sequentially increasing and decreasing the strength of the filament stimulus applied incrementally (minimum 0.04 g, maximum 4 g).

### 2.8. Radiological assessment

High-resolution X-ray images of the mediolateral plane of the ipsilateral femur were obtained at baseline and immediately following weekly behavioral assessments using a Faxitron MX-20 digital cabinet X-ray system (Faxitron/Bioptics, Wheeling, IL). Mice were lightly anesthetized with ketamine/xylazine (0.005 mL/g, 50/5 mg/kg, s.c.) and x-rayed at 30 kV for 12 seconds.

### 2.9. Statistical analysis

Postinjection response trajectories over time of rears and distance activity measures in sarcoma and naive animals were compared using linear mixed effects models (nlme package, R version 3.3.1^22,54^). To further quantify the divergence in responses on the outcome measures over time, 2-group *t* test comparisons were then conducted at days 7, 14, 21, 28, and 35 for each outcome variable. Von Frey skin sensitivity was computed as the difference between the von Frey scores for the 2 legs in each animal (score for the leg contralateral to the injection minus the score for the leg ipsilateral to the injection).

Receiver operating characteristic (ROC) analysis can be used to illustrate the performance of a binary classifier system as its discrimination threshold is varied. Receiver operating characteristic curves are created by plotting the true positive rate against the false positive rate at various threshold settings. The area under the ROC curve (also known as the concordance or c-statistic), along with the calculation of sensitivity and specificity, was used to determine the ability of each outcome measure to discriminate between the groups at each postinjection time point. Significant concordance was indicated if the 95% confidence interval (CI) of the c-statistic excluded chance-level concordance (0.50). Parallel analyses were conducted for activity outcome measures derived from the first exploratory hour (1200–1300 hours) and for activity measures across the first 3 nighttime hours (1900–2200 hours). All values are expressed as mean ± SEM. Significance level was set at *P* < 0.05.

## 3. Results

### 3.1. The progression of bone cancer

**Figure 1.**
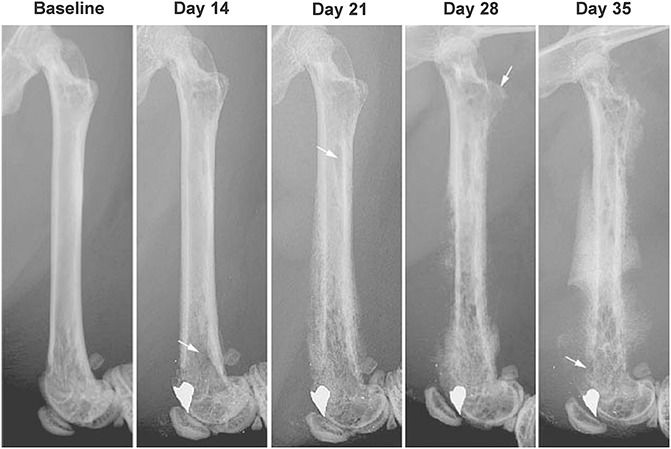
Radiographic images showing disease progression in a mouse model of bone cancer. Bone cancer was induced by drilling a 0.5-mm hole in the center of the trochlear groove of C3H/HeJ male mice (8–9 weeks old) and then injecting and confining 2472 sarcoma cells in the marrow space of the femur. Note that at day 14 post-tumor injection, x-rays show a noticeable tumor-induced bone remodeling which then becomes more severe at days 21, 28 and 35. Day 35 post-tumor injection was the last time point examined in this study.

Bone cancer was generated in mice by drilling a hole in cortical bone at the trochlear groove of the right femur, avoiding the cruciate ligaments and injecting osteolytic sarcoma cells into the intramedullary canal. To monitor disease progression, high-resolution radiographs were taken at weekly time points postcancer cell injection (Fig. 1). Within the tumor-bearing femur, bone destruction appears as radiolucent (darker) areas which are first evident in the distal end of the femur on day 14 post-tumor injection (arrow). With increasing time and disease progression, bone remodeling continues in the distal femur but also begins to involve both the midshaft and proximal areas of the femur. With disease progression, not only do the number and size of the focal radiolucencies increase but by day 35 post-tumor injection there is also new, ectopic bone formation as well as fracture of the cortical bone (arrow). While there is some heterogeneity between the extent of tumor-driven bone remodeling in the sarcoma bearing group, in terms of bone remodeling, animals within the naive group were always clearly distinguishable from the sarcoma bearing bones (Supplemental Figure 2, available online at http://links.lww.com/PR9/A7).

### 3.2. Horizontal locomotor activity decreases with bone cancer disease progression

**Figure 2.**
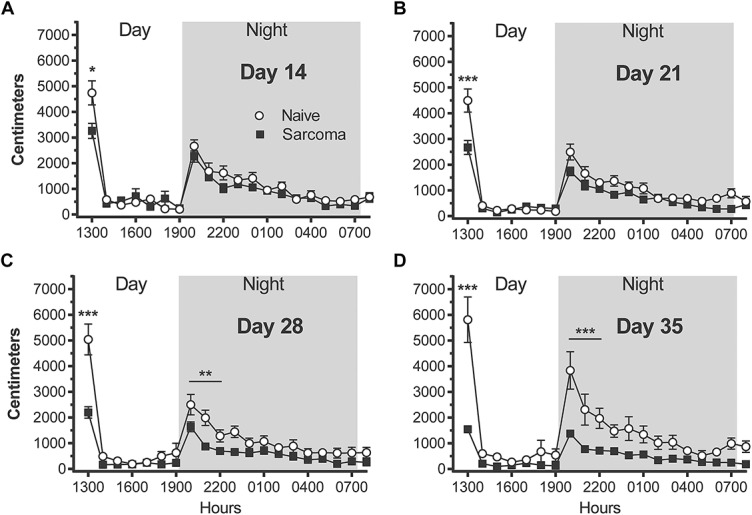
A reduction in horizontal locomotor activity is first observed at day 14 following tumor injection and continues to decline with disease progression. The activity of the mice was continuously recorded (A–D) for 20 hours, from 1200 (noon) to 0800 hours the following morning under a 12 hours light/dark cycle (0700–1900 hours light and 1900–0700 hours dark). Note that the times during the day/night where there is the largest differences between naive vs bone cancer animals are when the animals are normally the most active, ie, immediately following placement of the mice in the activity boxes (1200–1300 hours) and the first 3 hours of dark (1900–2200 hours). Significance is indicated by *, **, *** = *P* < 0.05, 0.01 and 0.001 respectively.

In mice, there are marked diurnal variations in horizontal activity (Fig. 2). When mice are first placed in the activity boxes during the light phase at noon (1200 hours), mice actively explore the novel environment for approximately 1 hour. By the end of this first daylight hour (1300 hours) most mice are at rest or asleep until the start of the dark phase (1900 hours) when mice again show significant increases in locomotor activity during the first 3 hours of night (1900–2200 hours). This period of increased horizontal activity during the daytime (1200–1300 hours) is consistent with the behavior of mice which rapidly explore a novel environment for potential threats,^14^ whereas the increased horizontal activity of the mice immediately after the room becomes dark at 1900 hours is consistent with the nocturnal nature of mice.

Postinjection response trajectories for nighttime horizontal locomotor activity over the 35-day observation interval were significantly different for sarcoma and naive groups (group × time interaction effect, *P* < 0.001). Beginning on day 14, post-tumor injection, there are significant differences in horizontal activity between naive vs tumor-bearing mice and with disease progression, the differences between the 2 groups increase with time (Fig. 2A–D). Sarcoma animals exhibited a significant reduction in horizontal distance traveled during the first exploratory hour (1200–1300 hours) at days 14, 21, 28, and 35 post-tumor injection (naive day 14 = 4741 ± 468 cm, sarcoma day 14 = 3257 ± 294 cm, naive day 21 = 4497 ± 453 cm, sarcoma day 21 = 2668 ± 274 cm, naive day 28 = 5037 ± 602 cm, sarcoma day 28 = 2198 ± 225 cm, naive day 35 = 5814 ± 886 cm, sarcoma day 35 = 1544 ± 120 cm). The sarcoma mice showed a significant decrease in peak locomotor activity during the first 3 hours of the dark phase of the light/dark cycle (1900–2200 hours), but the differences between naive and tumor-bearing animals during the first 3 nighttime hours was only significantly different at days 28 and 35 post-tumor injection (naive day 14 = 5971 ± 664 cm, sarcoma day 14 = 4802 ± 533 cm, naive day 21 = 5455 ± 649 cm, sarcoma day 21 = 3987 ± 412 cm, naive day 28 = 5768 ± 819 cm, sarcoma day 28 = 3197 ± 362 cm, naive day 35 = 8115 ± 1641 cm, sarcoma day 35 = 2852 ± 235 cm).

**Figure 3.**
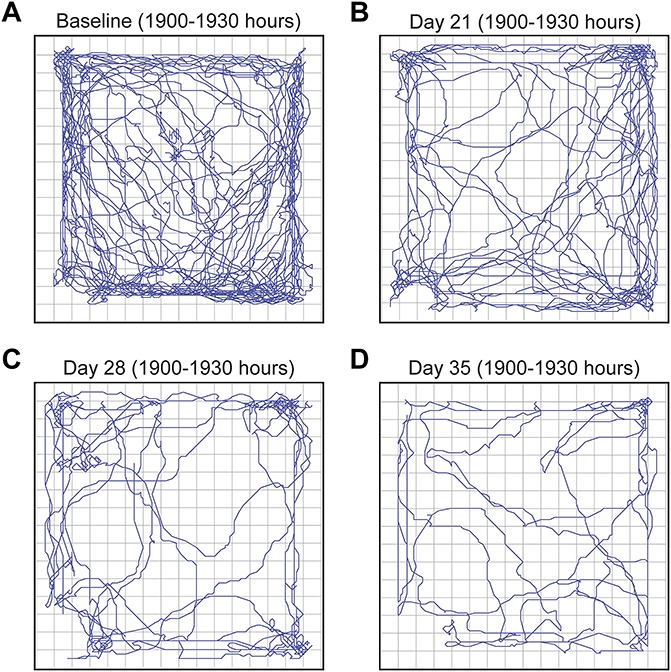
Representative tracings from a single animal showing the decline in nighttime horizontal activity with disease progression. These tracings were obtained from a single mouse at baseline (7 days prior to tumor injection) and at days 21, 28, and 35 post-tumor injection. The tracings were obtained over 30-minutes (1900–1930 hours) of nighttime, which is normally when mice display the greatest spontaneous activity. Note that horizontal activity continues to decline with disease progression.

Representative tracings of distance and the pattern of horizontal locomotor activity from a single mouse obtained over a 30-minute period during the dark phase (2000–2030 hours) are shown in Figure 3. Visually significant reductions in horizontal activity are apparent on days 21, 28, and 35 (Fig. 3B–D) in the activity boxes when compared with baseline (Fig. 3A).

### 3.3. Vertical rearing episodes decrease with disease progression

**Figure 4.**
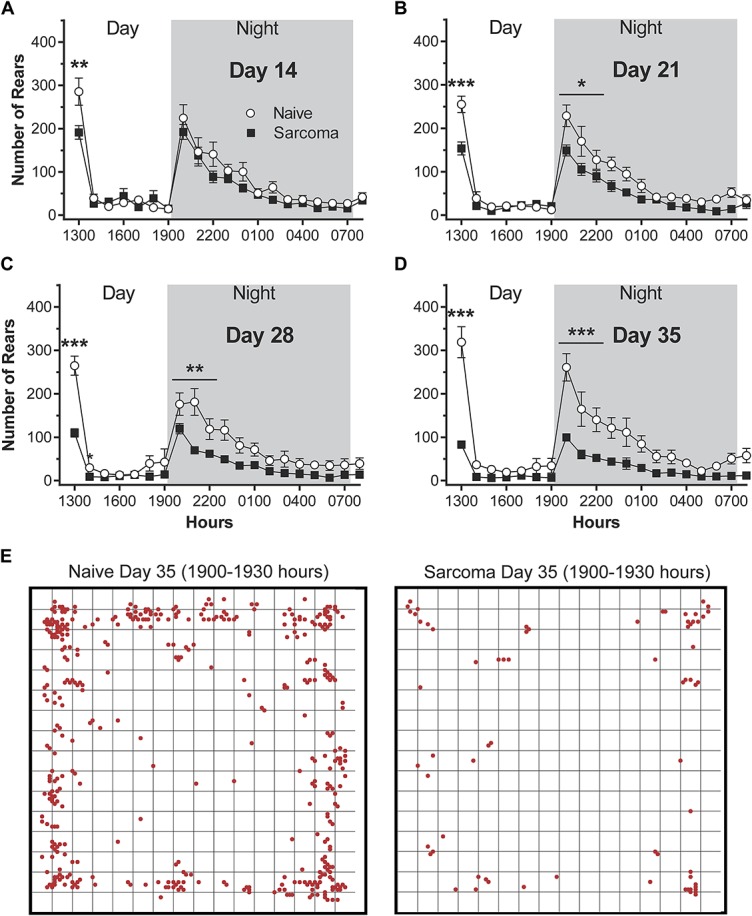
The number of hindlimb rearing episodes declines with bone cancer disease progression. These data were obtained by recording activity (A–D) for 20 hours, from 1200 (noon) to 0800 hours the following morning under a 12 hours light/dark cycle (0700–1900 hours light and 1900–0700 hours dark). Note that the times during the day/night where there are the largest differences between naive vs bone cancer animals are when the animals are normally the most active, ie, immediately following placement of the mice in the activity boxes (1200–1300 hours) and the first 3 hours of dark (1900–2200 hours). For (A–D), significant differences for the areas under the time-effect curves for the initial exploratory hour (1200–1300 hours) and the first 3 hours of night activity (1900–2200) are indicated by *, **, *** = *P* < 0.05, 0.01 and 0.001, respectively. Images in E are examples of both the number of episodes and where they occurred during 1900 to 1930 hours on day 35, naive and animals with bone cancer.

With bone cancer disease progression, there was a significant reduction in the number of vertical rearing episodes with disease progression (Fig. 4). A vertical rearing episode in mice requires loading^55^ and use of both the normal and tumor-bearing hindlimb and is thought to be an activity and exploratory behavior related to vigilance and escape.^14,41^

Postinjection response trajectories for vertical rearing episodes over the 35-day observation interval were significantly different for sarcoma and naive groups (group × time interaction effect, *P* < 0.001). Similar to what was observed in horizontal activity, beginning on day 14 post-tumor injection, there are significant differences between groups in vertical rearing episodes at the daytime exploratory hour (1200–1300 hours) but not the peak nighttime hours (1900–2100 hours) (Fig. 4A). Sarcoma animals exhibited a significant reduction in rearing episodes during the first exploratory hour (1200–1300 hours) at days 14, 21, 28, and 35 post-tumor injection (naive day 14 = 285 ± 31, sarcoma day 14 = 191 ± 16, naive day 21 = 255 ± 19, sarcoma day 21 = 154 ± 15, naive day 28 = 265 ± 22, sarcoma day 28 = 110 ± 10, naive day 35 = 319 ± 36, sarcoma day 35 = 83 ± 7), whereas these same mice showed a significant decrease in rearing episodes during hours 1900 to 2200 at days 21, 28, and 35 post-tumor injection (naive day 14 = 511 ± 76, sarcoma day 14 = 418 ± 44, naive day 21 = 526 ± 74, sarcoma day 21 = 344 ± 32, naive day 28 = 476 ± 65, sarcoma day 28 = 252 ± 24, naive day 35 = 565 ± 92, sarcoma day 35 = 212 ± 21) (Fig. 3B–D).

Representative tracings showing the numbers and places of the vertical rearing episodes obtained from a naive vs a tumor-bearing mouse at day 35 post-tumor injection are shown in Figure 4E. These tracings were obtained over a 30-minute period during the dark phase (2000–2030 hours). Note that there is a marked reduction in vertical rearing episodes in the tumor-bearing mouse.

### 3.4. Comparing bone cancer–induced hypersensitivity of the skin to changes in horizontal and vertical activity

**Figure 5.**
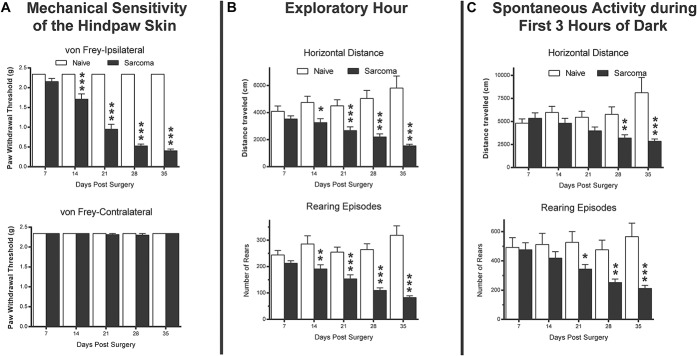
Comparison between cancer-induced bone pain changes in mechanical hypersensitivity of the skin, initial daytime exploratory activity and spontaneous nighttime activity. Note that at day 35 post-tumor injection mechanical hypersensitivity of the skin shows a greater than 80% decline, whereas initial daytime exploratory activity declines by approximately 60% and spontaneous nighttime activity declines by approximately 50%. Also note that mechanical hypersensitivity of the skin is highly significant (*P* < 0.001) by day 14 post-tumor injection, whereas initial daytime exploratory activity only reaches this level of significance at day 21 post-tumor injection and spontaneous nighttime activity only reaches this level of significance at day 35 post-tumor injection. Significance in differences between animals with bone cancer vs naive controls is indicated by *, **, *** = *P* < 0.05, 0.01 and 0.001, respectively. (A) *P* values represent the comparison of naive vs sarcoma on difference scores in skin sensitivity (contralateral minus ipsilateral for each animal) for each day.

In addition to monitoring both horizontal and vertical activity, mechanical hypersensitivity of the skin of the hind paw was also examined using von Frey testing (Fig. 5A). Similar to the changes observed for horizontal activity and vertical rearing episodes (Fig. 5B, C), development of bone cancer induces a marked mechanical hyperalgesia of the skin of the ipsilateral but not contralateral hind paw (Fig. 5A). This mechanical hyperalgesia is first evident on day 14 after tumor injection and continues to increase with disease progression. At day 14 post-tumor injection, which is the first time point post-tumor injection when visible tumor remodeling can be observed on X-rays, there is a large decrease (*P* = 0.001) in the mechanical hypersensitivity of the skin of the ipsilateral but not contralateral hind paw (Fig. 5A). At days 21, 28 and 35 post-tumor injection, the hypersensitivity of the ipsilateral skin increases so that by day 35 post-tumor injection the paw withdrawal threshold had declined from 2.34 to 0.41 g (Ipsilateral: naive day 7 = 2.34 ± 0 g, sarcoma day 7 = 2.15 ± 0.08 g, naive day 14 = 2.34 ± 0 g, sarcoma day 14 = 1.71 ± 0.13 g, naive day 21 = 2.34 ± 0 g, sarcoma day 21 = 0.95 ± 0.13 g, naive day 28 = 2.34 ± 0 g, sarcoma day 28 = 0.53 ± 0.05 g, naive day 35 = 2.34 ± 0 g, sarcoma day 35 = 0.41 ± 0.04 g).

**Figure 6.**
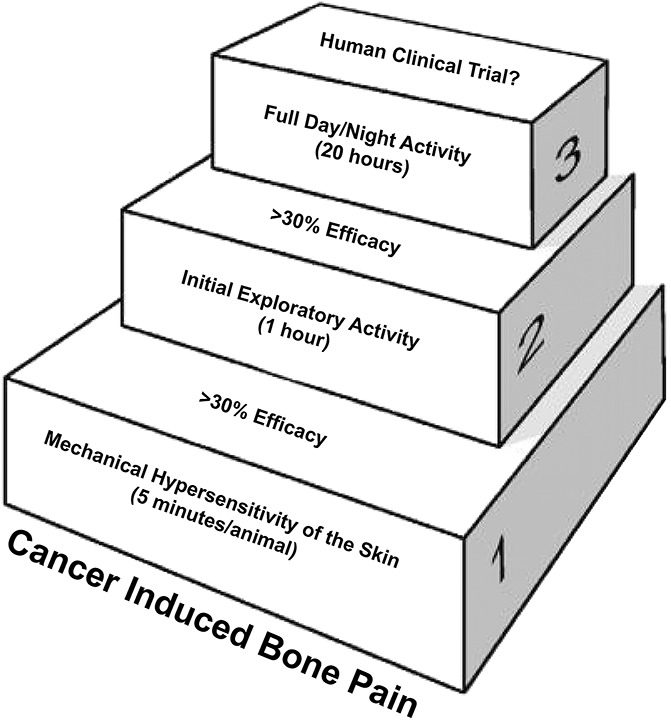
Schematic showing a stepwise approach for the preclinical assessment of novel analgesics targeting the relief of cancer-induced bone pain. The time for assessment of mechanical hypersensitivity of the skin is less than 5 min/1 mouse, initial daytime exploratory activity is 1 h/10 mice and spontaneous day/night activity is 20 h/10 mice.

Postinjection response trajectories for skin hypersensitivity over the 35-day observation interval were significantly different for sarcoma and naive groups (group × time interaction effect, *P* < 0.001). In comparing the changes in bone cancer–induced hypersensitivity of the skin to bone cancer–induced changes in horizontal or vertical activity, mechanical hypersensitivity of the skin is significantly different (*P* < 0.001) by day 14 post-tumor injection, whereas the initial 1-hour daytime exploratory activity only reaches this level of statistical significance at day 21 post-tumor injection and spontaneous nighttime activity only reaches this level of significance at day 35 post-tumor injection. Similarly, at day 35 post-tumor injection mechanical hypersensitivity of the skin shows a greater than 80% decline (Fig. 5A), whereas initial daytime exploratory activity declines by approximately 60% (Fig. 5B) and spontaneous nighttime activity declines by approximately 50% (Fig. 5C). Figure 6 outlines the behavioral tests that we employed in the present study.

### 3.5. Effect of surgery alone on horizontal activity and rearing episodes

To explore whether the surgery alone has a long-term effect on horizontal activity and rearing episodes, we compared naive baseline to days 1, 3, 7, and 12 postsurgery results. Animals had recovered from surgery and values were not significantly different from naive baselines (1900–2200 hours) for horizontal activity by day 7 postsurgery (naive baseline night = 5930 ± 547, day 7 night = 5289 ± 438, *P* = 0.99) (Supplemental Figure 3A, available online at http://links.lww.com/PR9/A7). As with horizontal activity, rearing episodes by day 7 postsurgery were not significantly different from naive baselines (naive baseline night = 618 ± 46, day 7 night = 483 ± 42, *P* = 0.06) (Supplemental Figure 3B, available online at http://links.lww.com/PR9/A7).

### 3.6. Discriminatory power of nighttime behavioral measures increases with disease progression

The ability of the behavioral indicators, measured in the initial 3 nighttime hours, to discriminate between sarcoma and naive animals was good early in the disease process and improved with disease progression. Skin hypersensitivity scores showed the best ability to discriminate between groups.

Sensitivity (true positive rate): skin hypersensitivity scores showed the highest sensitivity (ranging from 57% on day 14%–100% on day 35). For the activity measures, rears demonstrated highest sensitivity at the final observation time point (day 35). Sensitivity for behavioral measures (rears [53% on day 14, 86% on day 35], distance [60% on day 14, 71% on day 35]) were lower than the sensitivity of the skin hypersensitivity measure.

Specificity (true negative rate): skin hypersensitivity scores were 100% specific at all postinjection time points. Lower, but acceptable, specificity was observed for behavioral measures (rears, 74% on day 14, 78% on day 35), distance (74% on day 14, 87% on day 35).

Concordance (c-statistic): Only the skin hypersensitivity c-statistic was statistically significant at day 14 (c-statistic = 0.78, 95% CI = 0.68–0.89) and at every subsequent postinjection time point. Both activity measures demonstrated significant concordance at day 28 and day 35 (day 28: [rears 0.80, 95% CI = 0.65–0.94] [distance 0.75, 95% CI = 0.59–0.91]).

## 4. Discussion

### 4.1. Cancer-induced bone pain and its impact on the activity and functional status of the patient

Cancer-induced bone pain can occur either from a primary bone cancer such as an osteosarcoma or from a cancer that has metastasized to bone.^39^ Cancers that frequently metastasize to bone include the prostate, breast, renal, lung, and myeloma.^16^ The most common sites of tumor metastases are vertebrae, pelvis, long bones, and ribs.^39^ The major reason why CIBP can have a remarkably negative impact on a cancer patient's functional status and daily activity is that loading and use of the skeleton is required for most physical and social activities. Thus, if CIBP is present, normally innocuous strain and loading of the skeleton such as walking, exercise, sitting, or turning in bed can be perceived as highly painful, which greatly limits a patient's ability to remain active.^16^

In the past 2 decades, several preclinical rodent models of CIBP were developed to identify novel analgesic agents to control this pain.^29,61^ In many ways, these animal models have been remarkably successful in providing insight as to the mechanisms that drive CIBP and in the development of mechanism-based therapies to treat CIBP. In the past decade, newly approved agents that are now part of the standard care in treating CIBP include bisphosphonates, anti-RANKL antibodies, gabapentin/pregabalin, and radium 233 with others such as anti-NGF showing promise in phase II and III clinical trials.^25,45,50,65,66^ Most of these therapies were developed with the primary endpoint being the relief of CIBP. However, as patients with CIBP are living longer, a major goal in drug development for treating CIBP is now to not only relieve pain but also to improve the activity and functional status of the patient.^3,37,39,51^

### 4.2. Challenges in translating results from rodent models into human clinical trials

Currently, there are several major challenges to expanding our understanding of what drives CIBP and for behaviorally assessing pain, activity, and functional status in rodent models of CIBP. The first major problem in measuring pain in rodent preclinical models of CIBP is that in most cases the behaviors are performed during daylight hours (7 AM–7 PM) which is the time that rodents are normally least active or sleeping.^48,61^ By contrast, most human clinical trials examining the efficacy of an agent on relieving skeletal pain including CIBP usually focus on when the patient is normally awake,^25,51,65^ so it is not clear how one can readily translate results obtained from rodents during the time when the animal is least active vs assessments conducted when humans are normally most active.

A second major problem with current assessments of pain and functional status in models of CIBP is that nearly all rodent pain behaviors are evoked and not spontaneous. Currently, the most commonly used endpoint to measure CIBP in preclinical models is an increase in mechanical or thermal hypersensitivity of the skin of the hind paw as assessed by von Frey (mechanical testing) or the Hargreaves method (thermal).^11,28,31,49^ While peripheral and/or central sensitization may be driving CIBP-induced skin hypersensitivity,^2,34,57,61,72^ from the perspective of a patient with CIBP, evoked skin hyperalgesia is rarely the major pain complaint.^39^ Rather the major complaint of patients with CIBP is pain that arises on use of the skeleton that interferes with their ability to use and load their skeleton without significant movement evoked pain.^39^ Furthermore, it has recently been demonstrated that analgesics, such as anti-P2X3, that significantly attenuated CIBP-induced skin hypersensitivity, showed no efficacy in reducing the underlying skeletal pain, as measured by guarding, flinching and use of the tumor-bearing limb.^27^ By contrast, anti-NGF showed significant efficacy in attenuating both CIBP-induced skin hypersensitivity and CIBP-induced skeletal pain-related behaviors.^27^ Probably of equal importance, it is not clear how one would design, perform a power analysis, or determine what endpoints would be measured in a human CIBP trial that is based principally on the ability of an analgesic to relieve skin hypersensitivity in the rodent.

Third, even when “skeletal pain-related behaviors” such as limb guarding, flinching, weight bearing, or nocifensive behaviors are assessed by an observer during daylight hours,^27,42,56,61^ these behaviors are evoked in that the animal is removed from its home cage during their normal sleep period and their behaviors assessed during the least active period of the animal's day. Equally important, all observer-based assessments of pain are subject to unavoidable observer differences and bias. Thus, what is a guard or flinch to one observer may be scored differently by another observer. In addition, when the analgesic therapy under examination has either a large therapeutic effect or a noticeable side effect, it becomes even more difficult to fully remove observer bias.

In the present report, day/night horizontal and vertical activity was continuously monitored for a 20-hour period before the injection of tumor cells and then at days 7, 14, 21, 28 and 35 post-tumor injection. To monitor activity, the activity boxes continually measures spontaneous locomotion and hindlimb rearing. At day 7 post-tumor injection there were no significant changes in horizontal or vertical activity. However, at day 14 post-tumor injection there was a significant decline in initial exploratory activity and at day 21 there was a significant decline in spontaneous night activity, and this decline in activity continued to worsen with disease progression. In both naive and mice with cancer, activity was greatest during the first 60 minutes they were placed in the activity boxes (the initial daytime exploratory period) and for the first 3 hours following the onset of the dark cycle (peak night activity). Interestingly, it was when the animals were most active that one observes the greatest differences between the naive and CIBP animals. At other time periods during both the day and night, when the animals were less active and there was little spontaneous movement, very small or no significant differences were noted between naive animals vs animals with CIBP. Measures of sensitivity, specificity and concordance indicated that the ability of these behavioral indicators, measured in the initial 3 nighttime hours, to discriminate between sarcoma vs naive animals is good early in the disease process and improves with disease progression.

Together, the present data suggests that just as in dogs^6,7^ and humans^5,36,39^ with CIBP with disease progression, mice show a marked reduction in activity. The present data also emphasize the importance that, just as in humans with skeletal pain, focusing on measuring when the mouse is most active, greatly improves the ability to capture the reduction in activity in animals and humans with CIBP.^5–7,36,39^

While the present study has focused on the role pain plays in the decreased activity in mice with bone cancer, this reduction in activity may also be due to sickness behavior^68^ and/or fatigue.^10,59^ Previous studies have shown that cancer patients frequently suffer simultaneously from pain, sickness behavior and fatigue.^15^ Understanding the interplay between these 3 symptoms and how relief of one influences the others, as well as day/night activity, may be particularly beneficial in improving the overall functional status of cancer patients.^21^

### 4.3. Cancer-induced bone pain–induced changes in activity vs skin hypersensitivity

The present results clearly show CIBP not only produces marked changes in day/night activity but also significant changes in skin hypersensitivity in the ipsilateral but not contralateral hind paw. Interestingly, although all these changes appear to be driven by tumor cells confined to the ipsilateral femur, the peak changes from baseline were observed in skin hypersensitivity (80% change), followed by changes in daytime exploratory activity (60% change), followed by spontaneous night activity (50% change). Previous data have suggested that the skeletal pain-induced changes in skin hypersensitivity are due to central sensitization.^1,17,20,26,71,72^ However, other data suggests that maintenance of this central sensitization may require continual drive from peripheral nociceptors as the skin hypersensitivity immediately disappears when sensory nerves innervating the painful skeleton are blocked by application of a local anesthetic.^8,71^

Measurement of skin hypersensitivity is clearly easier and much less time consuming than the measurement of 20-hour day/night activity. However, it has been shown that some analgesics that relieve skeletal pain-induced skin hypersensitivity may not also relieve the underlying skeletal pain due to CIBP.^27,60^ Thus, measuring skin hypersensitivity alone may not provide clear insight into which analgesics will attenuate animal or human CIBP. If a major objective of preclinical research is to identify mechanism-based analgesics that will relieve pain and increase activity of humans with CIBP, then a stepwise measurement of skin hypersensitivity, initial daytime exploratory activity and then full 20-hour day/night activity in the same animal may provide a more translatable data set in terms of not only whether a therapy decreases pain but also increases the activity of the individual.

## Disclosures

P. W. Mantyh has served as a consultant and/or received research grants from Abbott (Abbott Park, IL), Adolor (Exton, PA), Array Biopharma (Boulder, CO), Johnson and Johnson (New Brunswick, NJ), Merck (White Plains, NY), Pfizer (New York, NY), Plexxikon (Berkeley, CA), Rinat (South San Francisco, CA), and Roche (Palo Alto, CA). The remaining authors have no conflicts of interest to declare.

This research was funded by NIH Grants CA154550, CA157449, and NS023970 to P. W. Mantyh.

## Appendix A. Supplemental Digital Content

Supplemental Digital Content associated with this article can be found online at http://links.lww.com/PR9/A7.
